# An Infantile Complication

**Published:** 1890-01-11

**Authors:** 


### AN INFANTILE COMPLICATION.

It certainly is remarkable that so little should be really known as to the causes and indications of so familiar a phenomenon as the green stools of young infants. The textbooks indeed tell us that it is owing to the change of bilirubin into biliverdin, which is clear enough, and that this change is often carried out further by exposure to the air; but some writers attribute this conversion to an acid reaction of the contents of the bowel. It is true that the motions, as tested by litmus paper, are frequently faintly acid, but the addition of acid concentrated or diluted to normal yellow stools makes them more intensely yellow, while it certainly never increases the greenness of the others. But Dr. Pfeiffor finds that normal stools treated with a potash or soda solution, and exposed to the air, acquire a deep green colour, and that green stools are always less acid than healthy ones, if not actually neutral. That they are not alkaline is due to the acidity of the secretions in the lower part of the bowel, and he attributes the green colour to the relative inability of the gastric juice, from its absolute or relative insufficiency, to act on the whole of the milk proteids, which then enter into alkaline combinations in the small intestine.

He noticed that when healthy infants consumed excessive quantities of milk the motions were always green, but yellow when the allowance was reduced. He then experimented on a healthy infant, giving it for alternating periods of two days dilute hydrochloric acid and carbonate of soda, and observed that under the acid treatment the motions were yellow, and under the alkaline green. From our own experience we had long since been convinced that carbonate of soda was not the panacea that some imagine for the digestive derangements of infants, and we have been in the habit of substituting a mixture of rhubarb and hydrochloric acid for the pulv. rhei. ca., or the pulv. rhei. idod. we formerly used for the treatment of green stools.

